# Behavioral and biochemical effects of a formulation of the traditional Chinese medicine, Kai-Xin-San, in fatigued rats

**DOI:** 10.3892/etm.2013.1256

**Published:** 2013-08-07

**Authors:** YUAN HU, YIN CAO, MING LIU, PING LIU, HONG CUI, GUO DAI-HONG

**Affiliations:** 1 Department of Clinical Pharmacology, Pharmacy Care Center, Chinese PLA General Hospital, Beijing 100853;; 2 Department of Psychology, Chinese PLA General Hospital, Beijing 100853;; 3 Tianjin University of Traditional Chinese Medicine, Tianjin 300193, P.R. China

**Keywords:** anti-fatigue effect, Kai-Xin-San, treadmill running test, traditional Chinese medicinal formula

## Abstract

The present study was designed to evaluate the anti-fatigue activity and the behavioral and biochemical effects of Kai-Xin-San (KXS) extracts on fatigued rats. The rats were randomly divided into six groups: untreated control (UC), running control (RC), RC treated with 13 mg/kg/day modafinil and RC treated with KXS at dosages of 125, 250 and 500 mg/kg/day, respectively. The treatments were administered orally. Anti-fatigue activity was assessed using the treadmill running test and serum biochemical parameters were determined using an autoanalyzer and commercially available kits. Furthermore, the standardization of the KXS extracts was ensured using a high-performance liquid chromatography (HPLC)-fingerprint. The extracts were shown to increase exhaustive running time in the treadmill running test and reverse the fatigue-induced reduction in hepatic/muscle glycogen and testosterone, in addition to reducing the lactate dehydrogenase (LDH), serum urea nitrogen (SUN), blood lactic acid (BLA) and β-endorphin levels in the serum of the fatigued rats. Moreover, the extracts enhanced superoxide dismutase (SOD) activity and decreased the malondialdehyde (MDA) levels in the serum of the fatigued rats. The results of this preliminary study indicated that KXS exhibits anti-fatigue activity. This was reflected in the effects on the biochemical markers for fatigue.

## Introduction

Fatigue is one of the most frequent and disabling nonmotor problems and results in a negative impact on cognitive and physical function. Chronic fatigue occurs with aging, depression, diabetes and Parkinson's disease and is one of the most common symptoms in primary care (1). Kai-Xin-San (KXS), a traditional Chinese medicinal formula for relieving psychological diseases, was originally prescribed by the famous Chinese doctor Sun Si-Miao and, at present, continues to be used to efficiently regulate central nervous system function, in addition to treating anxiety and depression (2). Compositionally, KXS contains four indigenous medicines: Ginseng (the root of *Panax ginseng* C.A. Meyer), Fu Ling (the white part of *Poria cocos* F.A. Wolf), Yuan Zhi (the root of *Polygala tenuifolia* Willd) and Shi Chang-Pu (the rhizome of *Acorus gramineus* Solander). Ginseng, as the principal active component in KXS, has been traditionally used for the development of physical strength, particularly in patients suffering from severe fatigue and hypoxia (3,4). The predominant active components of ginseng are ginseng saponins, also known as ginsenosides, polysaccharides, peptides, polyacetylenic alcohols and fatty acids (5).

In our previous study, KXS was shown to exert an antidepressant-like effect in mice, as assessed using the forced swim test (FST) and the tail suspension test (TST) (2). KXS may improve depressive behavior by increasing the expression of phospho-cAMP response element-binding protein (p-CREB) in CA1, CA3 and the dentate gyrus (DG) of the hippocampus, as shown in chronically stressed rats (2,3). Despite the popularity of KXS in the treatment of psychological diseases, there is no scientific evidence concerning the potential anti-fatigue effects of this formulation in animal models. Thus, in the current study, the anti-fatigue action of KXS extract was examined using the treadmill running test and the effects of KXS on behavioral and biochemical markers for fatigue were assessed. Specifically, the levels of hepatic and muscle glycogen, serum urea nitrogen (SUN) and lactic dehydrogenase (LDH) were evaluated.

## Material and methods

### Plant material and extraction

All medicines formulating KXS (*viz*. radix of *P. ginseng*, white part of *P. cocos*, radix of *P. tenuifolia* and rhizome of *A. gramineus*) were purchased from the Beijing Tong Ren Tang Group Co., Ltd. (Beijing, China) in 2009. The voucher specimens of the four plants, identified by Professor Ping Liu and registered under the numbers NU-90111, NU-82003, NU-79015 and NU-80617, respectively, were preserved at the pharmacy of the Herbarium of Traditional Chinese Medicinal (TCM), Chinese People's Liberation Army (PLA) General Hospital (Beijing, China). The total extract was prepared in accordance with our previous study (2). All the experiments were completed between April and May 2010.

### Standardization of KXS

The chemical fingerprinting of the extracts was analyzed. The chemicals used for the identification and quantification of three compounds in the KXS extract were tenuifoliside A, 1-O-(E)-benzoyl-[3-O-(E)-α-toluyl]-β-D-fructofuranosyl-(2→1)-[β-D-glucopyranosyl-(1→2)]-α-D-glucopyranoside and ginsenoside Rb1, respectively.

**Figure 1.**
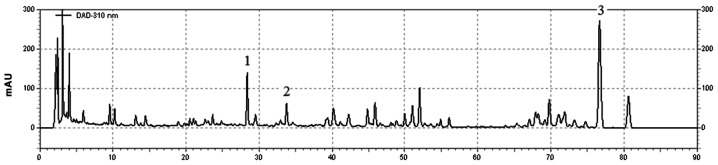
Chromatograms of the standard components of Kai-Xin-San (KXS). 1: tenuifoliside A [retention time (RT): 20.02 min); 2: 1-O-(E)-benzoyl-[3-O-(E)-α-toluyl]-β-D fructofuranosyl-(2→1)-[β-D-glucopyranosyl-(1→2)]-α-D-glucopyranoside (RT: 28.57 min); 3: ginsenoside Rb1 (RT: 55.41 min).

KXS extract (28 mg) was dissolved in 1 ml methanol and filtered through a 0.45 *μ*m syringe filter, prior to injection into the high-performance liquid chromatography (HPLC) system. The HPLC system was composed of an L-2200 Autosampler (Hitachi, Tokyo, Japan), an L-2130 pump (Hitachi), an ELSD 2000ES evaporative light scattering detector (Alltech Medical Systems, LLC, Cleveland, OH, USA) and an Agilent HC C18 (4.6×250.0 mm) column (Agilent Technologies, Santa Clara, CA, USA). A gradient solvent system of acetonitrile (A) and 0.65% ammonium acetate in water (B) was used as follows: 5%A/95%B (start), 15%A/85%B (8 min), 20%A/80%B (35 min), 28%A/72%B (60 min), 35%A/65%B (70 min), 100%A (74 min) and 5%A/95%B (75 min), at a flow rate of 1.0 ml/min (Fig. 1).

### Animals and drug administration

The experiments were performed using Sprague Dawley (SD) rats (weight, 180-220 g). The rats used in this study were cared for and treated humanely according to the ‘Guide for the Care and Use of Laboratory Animals’ of the Shanghai Institute of Material Medica. In this study, the mice were divided into six groups, as follows: untreated control (UC), running control (RC), RC treated with 13 mg/kg/day modafinil and RC treated with KXS at dosages of 125, 250 and 500 mg/kg/day, respectively. The 10-12 mice in each group were kept in a cage under standard laboratory conditions (at a temperature of 20±1°C and a 12-h light/dark cycle) with free access to food and water. All experiments were performed from 9:00 a.m. to 4:00 p.m. Modafinil, administered at a dose of 13 mg/kg, served as a positive control in the tests. The solutions of the tested samples were administered to the rats via gastric intubation at different dosages once a day at 9:00 a.m. The control groups received the same volume of the dosing vehicle (saline). The rats also received a single dose of the treatment 60 min prior to testing.

### Treadmill running protocols

The physical exercise load applied in the present study took the form of treadmill running on a motor-driven treadmill. The treatments were administered once a day for four weeks and 60 min prior to the running protocol. The rats of the RC and treatment groups were forced to run on a treadmill for 20 min once a day and the speed of the treadmill belt was gradually increased from 15 m/min to 35 m/min (slope of 15°). A failure to run caused the rat to slide off the moving belt and into a 15×15 cm electric shock grid that delivered 1.2 mA of current at 3 Hz. On the 30th day of the experiment, the time to exhaustion for treadmill running was determined for the exercise groups. Exhaustion was operationally defined as the third time a rat was no longer able to keep pace with the speed of the tread-mill belt and remained on a the electric shock grid for 2 sec rather than running. For each trial, the total time of exhaustive running was calculated and used as the best estimate of endurance running capacity.

### Blood and tissues collection

Following the behavioral test, rats were rapidly decapitated to obtain venous blood. The serum was separated by centrifugation at 1,800 × g and stored at −20ºC prior to being assayed. Brain, liver and muscle tissues were removed rapidly on the ice-plate. The tissues were washed with cold saline, blotted dry and stored at −80°C prior to being assayed.

### Measurement of biochemical parameters associated with fatigue

Levels of β-endorphin in the brain, hepatic and muscle glycogen, lactate dehydrogenase (LDH), serum urea nitrogen (SUN), blood lactic acid (BLA), testosterone and malondialde-hyde (MDA), and the activity of superoxide dismutase (SOD) in the serum were determined using commercially available kits from the Nanjing Jiancheng Bio-Company (Nanjing, China).

### Statistical analysis

Data are presented as the mean ± standard deviation. Overall differences according to the treatment were evaluated using one-way analysis of variance (ANOVA), while differences between groups were determined by analysis of variance and the Student's t-test (6). P<0.05 or P<0.01 was considered to indicate a statistically significant difference.

## Results

### HPLC fingerprint

Chromatograms of the KXS extract and its three major compounds are shown in Fig. 1. As the main component, the concentration of tenuifoliside A was 9.99±0.12 mg/g, while the concentrations of 1-O-(E)-benzoyl-[3-O-(E)-α-toluyl]-β-D-fructofuranosyl-(2→1)-[β-D-glucopyranosyl-(1→2)]-α-D-glucopyranoside and ginsenoside Rb1 were 16.71±0.16 and 5.79±0.04 mg/g, respectively.

### Effects of KXS in the treadmill running test

**Figure 2.**
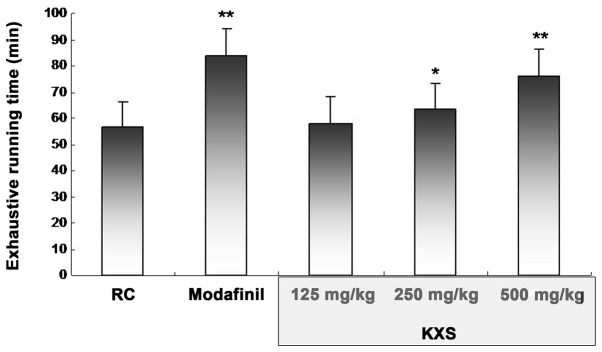
Effects of Kai-Xin-San (KXS) in the treadmill running test. Values are expressed as the mean ± standard deviation; n=10−12 in each group. *P<0.01 and ^**^P<0.05 compared with the running control (RC) group. RC group: rats were exposed to the treadmill running and treated with saline.

As shown in Fig. 2, in comparison with the RC group, 250 and 500 mg/kg KXS induced marked increases in exhaustive running time in the treadmill running test, with the most efficacious results demonstrated at a dose of 500 mg/kg (76.28±2.56 versus 56.40±3.61 min in the RC group, P<0.01)

### Effects of KXS on biochemical parameters of energy metabolism

As shown in Table I, exposure to the treadmill running test led to increases in the LDH, SUN, BLA and β-endorphin levels in the brain or serum, and decreased hepatic/muscle glycogen and testosterone levels compared with the UC group. All these effects were inhibited by 500 mg/kg KXS, which showed good effects on all biochemical parameters, especially β-endorphin and testosterone. Modafinil significantly affected only the levels of SUN, β-endorphin and testosterone.

### Effects of KXS on SOD activity and MDA level

**Figure 3.**
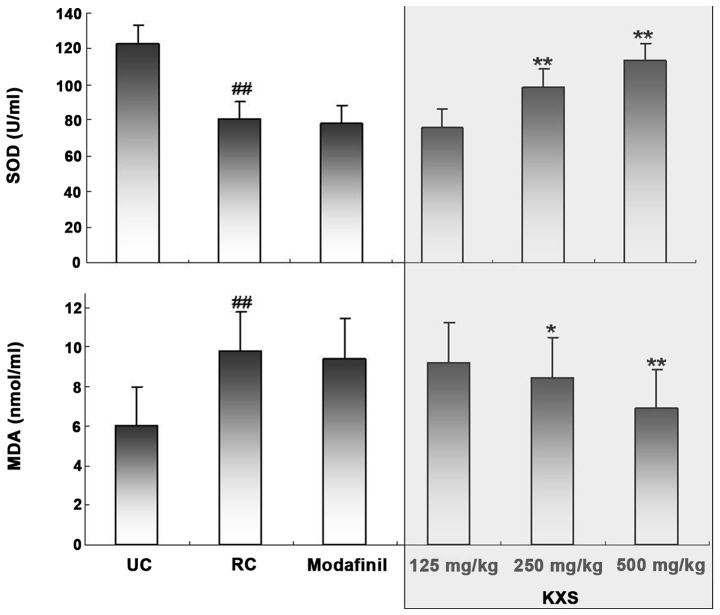
Effects of Kai-Xin-San (KXS) on superoxide dismutase (SOD) activity and malondialdehyde (MDA) levels in the treadmill running test. Values are expressed as the mean ± standard deviation; n=10−12 in each group. ^##^P<0.01 and ^#^P<0.05 compared with the untreated control (UC) group. ^*^P<0.01 and ^**^P<0.05 compared with the running control (RC) group. RC group: rats were exposed to the treadmill running and treated with saline; UC group: rats were not exposed to treadmill running and were treated with saline.

The serum total SOD activity and MDA level were measured at the end of the treadmill running test (Fig. 3). Exhaustive running induced a significant reduction in total SOD activity (P<0.01) and a significant increase in the level of MDA (P<0.01) compared with those in the UC group. KXS treatment dose-dependently increased the total SOD activity and decreased the MDA level; however, modafinil treatment did not demonstrate any significant effects on either variable.

## Discussion

Fatigue often occurs in aging, cancer, depression, HIV infection, multiple sclerosis and Parkinson's disease (7). However, there are very few pharmacological drugs or therapies available for the treatment of fatigue (1). Natural products may be used to improve athletic ability, postpone fatigue and accelerate the elimination of fatigue in human beings, with few side-effects (8). A direct measure of an anti-fatigue effect is the increase in exercise tolerance. Running to exhaustion is an experimental exercise model used to evaluate anti-fatigue effects and the endurance capacity of rodents, and gives a high reproducibility (9,10). The present study demonstrated that KXS exhibited anti-fatigue effects in the treadmill running test through the adjustment of one behavioral and several biochemical markers for fatigue. An HPLC-fingerprint was used as a tool to ensure the standardization of the KXS extract. Rats treated with KXS demonstrated significant increases in exhaustive running time. Moreover, the effects of KXS in the treadmill running test were accompanied by an attenuation of the fatigue-induced effects on the physiological markers relevant for fatigue.

One possible explanation for the anti-fatigue effect observed following treatment with KXS may involve glycogen mobilization during exercise. There are numerous biochemical parameters that are associated with glycogen mobilization during exercise and fatigue. LDH catalyzes the interconversion of pyruvate and lactate, with levels increasing during exercise (11). Serum urea nitrogen, which is the product of energy metabolism when moving, is a sensitive index used to evaluate the bearing capability when human bodies suffer from a physical load. The less an animal is adapted to exercise, the more LDH and SUN levels increase (12). Mild androgen deficiency may account for increased fatigue and testosterone replacement has been shown to improve these abnormalities (13,5). In addition, a lack of a β-endorphin response has been demonstrated to be beneficial to endurance exercise (14). Moreover, the liver/muscle glycogen and BLA levels are also sensitive parameters associated with fatigue. The depletion of these stores of glycogen may be detrimental to an animal's capacity to function and a high level of lactic acid may lead to a reduction in the pH of the muscle tissue and the blood during high-intensity exercise (15).

These seven biochemical parameters exhibited significant differences in this exhaustive running test. The high and middle-doses of KXS reduced the levels of LDH, SUN, BLA and β-endorphin and increased the levels of hepatic/muscle glycogen and testosterone in fatigued rats. The results showed that the anti-fatigue activity of KXS may have been correlated with an improvement in the metabolic control of exercise and the activation of energy metabolism. This was consistent with the study by Wang *et al* (5). Another possible explanation for the anti-fatigue effect observed following treatment with KXS may involve its antioxidant activity during exercise. In a previous assay, enhanced oxidative stress was observed in a rat model of fatigue and depression, predominantly expressed by a significant increase in the MDA level and a significant reduction in SOD activity (16). The present data were consistent with these studies. Our results also indicate that KXS is able to increase SOD activity and reduce lipid oxidation in exhaustive running models, thereby showing marked antioxidant effects. The results suggest that the anti-fatigue effect of KXS most likely occurred through the protection of the corpuscular membrane by the prevention of lipid oxidation.

In conclusion, the present study demonstrated the anti-fatigue activity of the KXS formulation in the treadmill running test. The biochemical mechanisms of the anti-fatigue effects demonstrated by KXS may be due to its antioxidant activity and the improvement in the metabolic control of exercise and the activation of energy metabolism. Further studies regarding the isolation of the major bioactive components of KXS responsible for the observed effects and the precise site and the mechanism of action are required.

## Figures and Tables

**Table I.** Effects of Kai-Xin-San (KXS) on the biochemical parameters involved in energy metabolism.

Group	Dose (mg/kg)	LDH (U/l)	SUN (nmol/l)	BLA (nmol/l)	β-endorphin (ng/l)	Testosterone (nmol/l)	Hepatic glycogen (mg/g)	Muscle glycogen (mg/g)
UC	-	2600.86±200.90	3.39±0.16	5.71±0.15	451.61±31.41	16.07±1.25	30.10±1.45	1.41±0.11
RC	-	3140.52±170.43^a^	7.26±0.55^a^	10.33±0.95^a^	582.06±42.79^a^	14.28±0.58^b^	20.71±2.05^a^	1.15±0.10^a^
Modafinil	13	3184.48±275.85	6.30±0.24^d^	9.98±0.76	445.99±29.52^d^	15.81±0.65^d^	20.13±2.86	1.22±0.16
KXS	125	2434.07±250.78^d^	6.54±0.53^c^	7.36±0.59^d^	456.11±26.90	16.96±0.53^d^	23.64±0.82^d^	1.44±0.16^d^
KXS	250	3119.83±180.14	7.21±0.28	10.64±0.70	537.07±26.32	16.35±0.55^d^	18.83±1.95	1.32±0.34
KXS	500	2983.62±228.89	6.75±0.20	8.56±1.18^d^	431.37±20.24^d^	17.35±0.72^d^	23.17±1.96^c^	1.52±0.11^d^

Values are expressed as the mean ± standard deviation; n=10-12 in each group.

a P<0.01 and

b P<0.05 compared with the untreated control (UC) group.

c P<0.01 and

d P<0.05 compared with the running control (RC) group. RC group: rats were exposed to treadmill running and treated with saline; UC group: rats were not exposed to the treadmill running and were treated with saline. LDH, lactate dehydrogenase; SUN, serum urea nitrogen; BLA, blood lactic acid.
